# Green Synthesis of Tin Oxide (SnO_2_) Nanoparticles Using Ginger Extracts for Photocatalytic Degradation of Organic Dyes in Wastewater

**DOI:** 10.1007/s12010-025-05293-2

**Published:** 2025-06-27

**Authors:** Yuvana Sivarajan, Khairul Anwar Ishak, Jamilah Syafawati Yaacob

**Affiliations:** 1 Institute of Biological Sciences, Faculty of Science, Universiti Malaya, 50603 Kuala Lumpur, Malaysia; 2 Institute of Systems Biology, Universiti Kebangsaan Malaysia, 43600 UKM Bangi, Selangor Malaysia; 3 Centre for Research in Biotechnology for Agriculture (CEBAR), Institute of Biological Sciences, Faculty of Science, Universiti Malaya, 50603 Kuala Lumpur, Malaysia

**Keywords:** Tin oxide nanoparticles, Ginger extract, Photocatalytic degradation, Dyes, Water pollution

## Abstract

One of the main issues that has been receiving great attention globally is the pollution of water resources with dyes, as the chemicals present in them are carcinogenic and harmful to aquatic organisms and humans. Metal oxide nanoparticles synthesized using plant extracts have recently emerged as the best option to address this problem as they provide an eco-friendly and inexpensive approach for a clean water supply. This study describes the photocatalytic degradation of four different types of dyes—methylene blue (MB), rhodamine B (RB), Eriochrome black-T (EB), and methyl orange (MO) using tin oxide nanoparticles (SnO_2_ NPs) synthesized with ginger (*Zingiber officinale*) extract. From the results, the SnO_2_ NPs play a remarkable role in degrading all the dyes even though they require a longer time to reach maximum degradation. This study also describes the biosynthetic preparation of SnO_2_ NPs, which have a  tetragonal structure, prepared using the optimum parameters of 0.02 g mL^−1^ of ginger extract, 0.5 M of tin (II) chloride dihydrate (SnCl_2_.H_2_O), and reaction pH of 5. SnO_2_ NPs were characterized using powder X-ray diffraction (XRD) to evaluate their crystallinity and composition, Fourier transform infrared (FTIR) to identify the changes in functional groups of SnO_2_ NPs, and ultraviolet–visible (UV–Vis) spectroscopy to analyze the optical properties of SnO_2_ NPs through band gap calculation. The photocatalytic activity of SnO_2_ NPs was then studied through their efficiency in degrading the four dyes. A toxicity test was also carried out in this study using brine shrimp (*Artemia salina*) to ensure the safety of the synthesized SnO_2_ NPs made with ginger extract for use in aquatic environments to degrade pollutants. The positive results obtained indicate their potential as a good photocatalyst for water remediation.

**Supplementary Information:**

The online version contains supplementary material available at 10.1007/s12010-025-05293-2.

## Introduction

Approximately 80% of municipal (domestic and commercial) and industrial wastewater are discharged into the environment without pre-treatment, causing adverse effects to the ecosystems [58]. This raises concern about the widespread problem of water pollution, as it affects both human and environmental health. For example, about 1.3 trillion gallons of wastewater from textile industries are discharged  into streams and rivers, containing toxic chemicals including synthetic dyes and heavy metals [100]. The increased presence of dyes in water bodies would affect aquatic photosynthesis due to reduced sunlight penetration. As a result, the biological and chemical oxygen demand increases, leading to the death of aquatic life [41]. Furthermore, consumption of dye-polluted water can affect humans by causing health complications such as cancer, skin diseases, and allergies [74]. In recent years, many initiatives for effluent treatment such as electrolytic method [107], electron beam treatment [40], activated carbon [101], and photocatalysis [8] have been taken to address this issue. Photocatalysis, also known as photocatalytic degradation, is one of the most recommended approaches due to its ecofriendly, economical, and sustainable features.

In photocatalysis, light energy is used to activate chemical reactions [90]. Photocatalysts harness light energy to generate hydroxyl radicals, which can oxidize synthetic dyes, thus reducing their toxicity. Several photoactive metal oxides such as  iron (III) oxide, titanium oxide, zinc oxide, vanadium oxide, and tin oxide are studied and used as photocatalysts for wastewater treatment [79]. Nowadays, tin oxide nanoparticles (SnO_2_ NPs) have gained significant attention as a semiconductor oxide and photocatalyst in the process of breaking down organic compounds [51]. They have a strong oxidizing property as well as high optical transparency due to  their large band gap of around 3.6 eV [51], which corresponds to activation by photons with a wavelength of 350 nm (UV-A range) [7]. They also have high electron mobility (100 to 200 cm^2^ V ⁻^1^ s⁻^1^) that speeds up the photogenerated electron transport [95]. SnO_2_ NPs can be synthesized through numerous techniques such as hydrothermal synthesis, laser ablation, and flame spray pyrolysis; however, in recent years, the bio-based green synthesis method has garnered considerable attention [42]. This is because of the low-cost, straightforward, and ecofriendly production, as they avoid the use of toxic chemicals and harsh conditions, such as extreme temperatures, pressures, or high acidity and alkalinity [77]. 

The green synthesis method utilizes plant extracts that are rich in active phytochemical constituents such as flavonoids, phenols, and terpenoids, which act as stabilizing and reducing agents to convert metal ions into their oxide form [104]. For instance, ginger is abundant in various active phytochemicals, including flavonoids, alkaloids, antioxidants, terpenoids, and amino acids, which can usually be found in the leaves, fruits , peels, and seeds of plants. Moreover, plant-based green synthesis enhances the stability of nanoparticles in terms of shape and size, and it produces a higher yield of nanoparticles compared to other chemical and physical methods [46].

Despite its ecofriendly and economical features, limited published literature can be found on bio-based green synthesis of SnO_2_ NPs [85]. This study investigates the potential of bio-based green synthesis of SnO_2_ NPs using flavonoids, particularly quercetin, in ginger (*Zingiber officinale*), which acts as a reducing agent to efficiently convert Sn^2+^ into SnO_2_. This study also explores the synergistic effect of variables in synthesizing SnO_2_ NPs using response surface methodology (RSM). X-ray diffraction (XRD) analysis, Fourier transform infrared (FTIR) spectroscopy, and ultraviolet–visible (UV–Vis) spectroscopy were employed to characterize the physicochemical properties of the synthesized SnO_2_ NPs. Furthermore, the photocatalytic activity of the synthesized SnO_2_ NPs in degrading dyes—methylene blue, rhodamine B, Eriochrome Black-T, and methyl orange—all of which are common water pollutants, was examined. A toxicity test using brine shrimp (*Artemia salina*) was also conducted to determine the  safety of the synthesized SnO_2_ NPs in aquatic environments. This study aims to provide valuable insights into plant-based green synthesis of SnO_2_ NPs using ginger extract and its potential as an applicable substitute for a more affordable and environmentally friendly wastewater treatment agent.

## Methodology

### Materials

Pure ginger (*Zingiber officinale*) powder (each containing 200 g) was purchased from a local pharmacy, Multicare. Chemicals such as methylene blue (Sigma-Aldrich, 100 g), rhodamine B (R&M, 25 g), Eriochrome black-T (Sigma-Aldrich, 25 g), and methyl orange (Chemiz, 25 g) dyes as well as tin (II) chloride dihydrate (SnCl_2_.2H_2_O) (250 g) were purchased from a local supplier. Ultrapure water (UPW) was used throughout the experiment, but to dissolve the dyes, distilled water (dH_2_O) was used. Seventy percent methanol, 10% aluminium chloride hexahydrate (AlCl_3_.6H_2_O), 1 M sodium acetate (NaC_2_H_3_O_2._3H_2_O), and a stock solution of 1 mg mL^−1^ quercetin were used in the determination of total flavonoid content (TFC). Brine shrimp (*Artemia salina*) eggs, sodium chloride (NaCl), and potassium dichromate (K_2_Cr_2_O_7_) were used in the toxicity analysis.

### Preparation of Aqueous Ginger (*Zingiber officinale*) Extract

The stock of ginger (*Zingiber officinale*) extract was prepared prior to photocatalyst synthesis. To this end, 200 g of ginger powder was stirred in 4000 mL of UPW using a glass rod and the mixture was then sonicated in an ultrasonic water bath (40 kHz, 70% of 500 W) for 30 min to enhance the extraction process. The solution was then filtered through Whatman filter paper 1 (Qualitative, 55 nm) using a filter vacuum. The filtrate (ginger extract) was collected and transferred into 4 Schott bottles of 1 L each. They were stored in the freezer at − 20 °C for further use. The concentration of ginger extract was determined as 0.05 g mL^−1^ through a simple drying process.

### Determination of Total Flavonoid Content (TFC)

The total flavonoid content (TFC) of ginger extract was determined using aluminium colorimetric method [106] with slight modifications. Fifteen microliters of 20 mg mL^−1^ ginger extract was mixed with 45 µL of 70% methanol, 3 µL of 10% aluminium chloride hexahydrate, 3 µL of 1 M sodium acetate, and 84 µL of UPW in a 96 well plate. The mixture was incubated at room temperature for 40 min, and the absorbance was measured at 415 nm in triplicate using a microplate spectrophotometer (EPOCH, Biotek) at INFRA Analysis Laboratory, Universiti Malaya. A quercetin standard curve (Supplementary Figure S1) with concentrations ranging from 0.10 to 0.60 mg mL^−1^ was prepared to calculate the TFC of ginger extracts. The results were expressed as quercetin equivalent in mg/g of dry extract (mgQE/g DE). The calculation for the TFC of ginger extract is shown in Eq. 1:

1 $$C=cV/m$$

where $C$ is TFC in mgQE/g dry extract, $c$ is the concentration of quercetin obtained from calibration curve (mg mL^−1^), $V$ is the volume of extract (mL), and $m$ is the mass of extract (g).

### Screening Experiments—One-Factor-at-a-Time (OFAT) Method

Preliminary experiments were conducted to determine the single factor effect on the yield of tin oxide nanoparticles (SnO_2_ NPs). Three factors, (i) ginger extract concentration, (ii) SnCl_2_.2H_2_O concentration, and (iii) pH of reaction, were varied in this experiment, as follows:

For the first variable, 6 different ginger extract concentrations (0.00, 0.01, 0.02, 0.03, 0.04, and 0.05 g mL^−1^) were tested, with the concentration of SnCl_2_.2H_2_O fixed at an estimated optimum of 0.1 M [1, 15] and the pH of reaction set to an estimated optimum of 5 (Abdo et al., 2021). The ginger extract concentration that produced the highest yield was then used to conduct screening for the second variable, i.e., SnCl_2_.2H_2_O concentration, with a range of 0.1–0.5 M while keeping the pH of reaction fixed at 5. After determining the optimal concentrations of ginger extract and SnCl_2_.2H_2_O, screening for the third factor, i.e., pH of reaction, was conducted at pH range of 3–7.

In all the experiments, the yield of SnO_2_ NPs was determined by harvesting the precipitate through centrifugation, followed by oven-drying it overnight at 80 °C and calcination in a furnace at 500 °C for 3 h. Prior to these steps, the precipitate was rinsed with methanol and centrifuged twice. The reaction time and temperature were fixed at 1 h and 65 °C, respectively, with the solution continuously stirred at a constant speed of 100 rpm throughout the experiment. UPW was used as the solvent for ginger extract, whereas methanol was used for SnCl_2_.2H_2_O. The pH of the reaction was adjusted using 1 M NaOH and 1 M HCl. The total volume of the reaction medium was 25 mL, consisting of 20 mL of ginger extract and 5 mL of SnCl_2_.2H_2_O. All experiments for each variable were conducted in triplicates.

### Evaluation of Three Variables Relationship Through Box-Behnken Design (BBD)

The effects of three selected independent variables (ginger extract concentration, SnCl_2_.2H_2_O concentration and pH of reaction) were investigated using the Box-Behnken design (BBD). The response variable in this experiment was the yield of SnO_2_. The range values for each variable were determined from the preliminary experiments. Randomized run orders for SnO_2_ NPs synthesis and all statistical analyses were performed using Minitab software. From the analysis, 3D surface plots were generated to examine the relationship between the variables and identify the optimal value. A regression equation in uncoded units, which can be used to calculate the theoretical yield, was also derived from the analysis. The percentage difference (percent error) between the experimental and theoretical yields was determined using Eq. 2 as shown below.

2 $$Percent\;error=\frac{\left(\text{Experimental value}-\text{Theoretical value}\right)}{\text{Theoretical value }}\times 100\%$$

### Characterization of Tin Oxide Nanoparticles (SnO_2_ NPs)

The SnO_2_ NPs were subjected to powder X-ray diffraction (XRD) analysis to evaluate their crystal structure (crystallinity) and composition using an X-ray powder diffractometer (Rigaku Miniflex). Data were recorded in the range of 2θ values from 10 to 90°, with a scanning rate of 4° min^−1^, using Cu-Kα radiation of *λ*_max_ = 1.54 Å [66]. Furthermore, the functional groups in the purified dried SnO_2_ NPs sample were analyzed using Fourier transform infrared (FTIR) spectroscopy (Perkin Elmer Spectrum 400) in the wavenumber range of 500–4000 cm^−1^, with a resolution of 4 cm^−1^, using potassium bromide pellets in diffuse reflectance mode. The FTIR spectrum was recorded to identify any changes in functional groups of the biosynthesized SnO_2_ NPs. Ultraviolet–visible (UV–Vis) spectroscopy was also performed using a Perkin Elmer Lambda 35 UV–Vis spectrophotometer at room temperature to analyze the optical properties of synthesized SnO_2_ NPs, particularly through the band gap derived from the absorption spectrum.

### Photocatalytic Activity of Tin Oxide Nanoparticles (SnO_2_ NPs)

Photocatalytic degradation of four different dyes—methylene blue (MB), rhodamine B (RB), Eriochrome black-T (EB), and methyl orange (MO)—was performed using the synthesized SnO_2_ NPs as the photocatalyst under UV light irradiation. The decolorization of the dyes was monitored using a UV–Vis spectrophotometer by tracking changes in the absorbance spectra at 20-min intervals, within the range of 300–700 nm. The concentration changes of MB, RB, EB, and MO dyes were determined and recorded by measuring their peak absorbance values at 664, 554, 489, and 464 nm, respectively. The standard curve for each dye is presented in Supplementary Figure S2.

In all the experiments, 60 mL of MB, RB, EB, and MO solutions were prepared at concentration of 10, 5, 10, and 10 ppm, respectively. The dye solutions were stirred vigorously for 30 min in the dark to establish adsorption–desorption equilibrium. Subsequently, 0.02 g of SnO_2_ NPs was added into 60 mL dye solution, which was then exposed directly to a UV light source (approximately 7 cm from the meniscus of each solution to the lamp) for 20 min with continuous stirring. During the experiment, 3 mL of each dye solution was sampled and centrifuged, and 1 mL of the supernatant was transferred into separate plastic cuvettes for absorbance measurement. The UV light was switched off to temporarily halt the photocatalytic degradation during sampling and measurement. After measurement, each dye solution was mixed with the photocatalyst in the same centrifuge tube and poured back into the original solution. This process was repeated five times, resulting in a total reaction duration of 100 min. The dye degradation efficiency (DE) catalyzed by the SnO_2_ NPs was calculated according to Eq. 3 [5, 11] as follows:

3 $$\begin{array}{c}\text{Degradation efficiency }\left(\text{DE};{\%}\right)=\left[\left({A}_{0}-{A}_{t}\right)/{A}_{0}\right]/\times 100\%\\ or\\ =\left[\left({C}_{0}-{C}_{t}\right)/{C}_{0}\right]\times 100{\%}\end{array}$$

where *A*_*0*_ is the initial dye absorbance, *A*_*t*_ is the dye absorbance after UV irradiation (at variable time, *t* = *t*), *C*_*0*_ is the initial dye concentration, and *C*_*t*_ is the dye concentration after UV irradiation (at variable time, *t* = *t*).

### Toxicity Effect of Tin Oxide Nanoparticles (SnO_2_ NPs) in Comparison with Standard, Potassium Dichromate (K_2_Cr_2_O_7_) on Brine Shrimp (*Artemia salina*)

One gram of brine shrimp (*Artemia salina*) eggs was placed in a container containing artificial seawater (0.038 g mL^−1^) for hatching. The process was conducted at room temperature under continuous illumination and aeration for 48 h. Stock solutions of SnO_2_ NPs and K_2_Cr_2_O_7_ (400 and 100 µg mL^−1^, respectively) were prepared, and dilution series for both solutions were made as detailed in Supplementary Tables S1 and S2. Five milliliters of each diluted solution (including control) was placed in each well as the medium for the toxicity test. Ten active *Artemia salina* nauplii were transferred into each well using a fine paintbrush. After 24 h, each well of the plates was observed under microscope to assess the condition of the *Artemia salina* nauplii in the presence of SnO_2_ NPs and K_2_Cr_2_O_7_. All experiments were conducted in triplicate for both the test sample, SnO_2_ NPs, and standard, K_2_Cr_2_O_7_.

The data was recorded based on the number of *Artemia salina* nauplii that survived per well. They were considered dead when no appendage movement was observed within 10 s. The percentage mortality of *Artemia salina* nauplii was calculated using Eq. 4 as follows:

4 $$\%\;Mortality=\;\frac{\mathrm{Number}\;\mathrm{of}\;\mathrm{dead}\;Artemia\;salina\;\mathrm{nauplii}}{\mathrm{Initial}\;\mathrm{number}\;\mathrm{of}\;\mathrm{alive}\;Artemia\;salina\;\mathrm{nauplii}}\times100\%$$

The results were interpreted, tabulated, and presented in graph form. The lethal concentration (LC_50_) of SnO_2_ NPs on *Artemia salina* was determined from the plotted mortality-versus-concentration curve, where the LC_50_ value corresponds to the concentration on the *x*-axis at which the mortality percentage reaches 50%. The test sample, SnO_2_ NPs, is considered non-toxic and safe for use in aquatic environment if the survival rate is ≥ 90% [31].

## Results and Discussion

### Preliminary Study on SnO_2_ NPs Synthesis

**Fig. 1:**
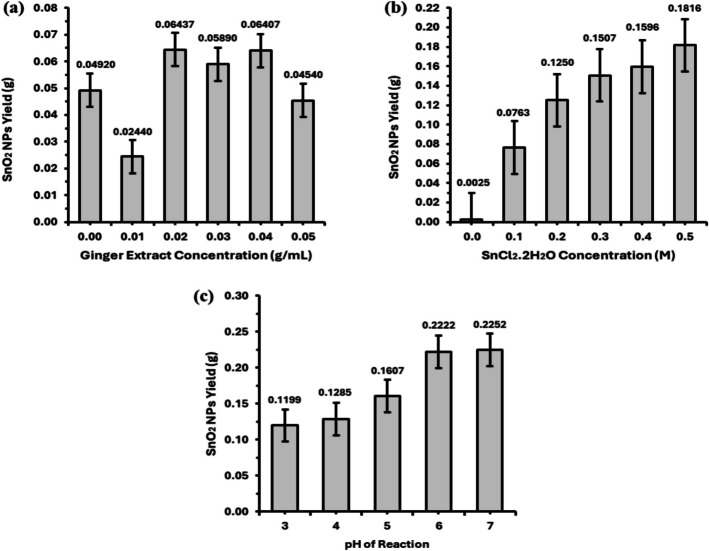
Effects of **a** ginger extract concentration, **b** SnCl_2_.H_2_O concentration, and **c** pH of reaction on SnO_2_ NPs yield

The effect of ginger extract concentration on the yield of SnO_2_ NPs is shown in Fig. 1a. The highest yield of SnO_2_ NPs was observed within the ginger extract concentration range of 0.02–0.04 g mL^−1^, with an average yield of approximately 0.06 g. However, as the ginger extract concentration was increased to 0.5 g mL^−1^, the SnO_2_ NPs yield decreased by about 29%. A higher amount of  reducing agent may shift the reaction equilibrium away from the desired tin oxide, potentially leading to incomplete oxidation or the formation of unwanted products [29]. Among the tested concentrations, 0.03 g mL^−1^ of ginger extract produced the most consistent yield of SnO_2_ NPs as compared to 0.02 and 0.04 g mL^−1^, which produced higher but inconsistent yields. Therefore, this concentration was selected for subsequent preliminary tests of other variables—SnCl_2_.H_2_O concentration and pH of reaction.

In the second preliminary experiment, SnO_2_ NPs yield increased proportionally to the concentration of SnCl_2_.2H_2_O (Fig. 1b). The highest yield of SnO_2_ NPs (0.1816 g) was obtained at the highest concentration of SnCl_2_.2H_2_O (0.5 M). Nevertheless, the SnO_2_ NPs yield obtained was not consistent when 0.5 M of SnCl_2_.2H_2_O was used as compared to 0.4 M. Therefore, 0.4 M of SnCl_2_.2H_2_O was considered the limit and thus used for the following third preliminary test, which was the pH of reaction.

In the third preliminary experiment, high yields of SnO_2_ NPs were obtained at pH 6 and 7, as shown in Fig. 1c. The yields of SnO_2_ NPs at these two pH values were 0.2222 and 0.2252 g, respectively. This shows that SnO_2_ NPs synthesized through this bio-based green synthesis using ginger extract is indeed eco-friendly, as this process of synthesis takes place at a neutral pH condition. In support of this study, a similar result [38] was obtained on other metals like silver, which indicates that at pH 6.7 and 7.7, many functional groups are available for silver binding (to facilitate a higher number of Ag ions to bind), and this was said to eventually form a large number of nanoparticles with smaller diameters.

### Study of SnO_2_ NPs Production Using Box-Behnken Design (BBD)

#### Statistical Analysis and Model Fitting

Based on the results of preliminary experiments on SnO_2_ NPs yield, the range values of each variable for the response surface methodology (RSM) were determined and classified as shown in Table 1. Randomized run orders of the independent variables in Table 1 were generated using Minitab software, and the order list is shown in Table 2. The corresponding yield of SnO_2_ NPs (response variable) for each run is tabulated in Table 2.

**Table 1:** Experimental points obtained for each of the three variables from BBD

Independent variables	Levels		
	Lowest	Middle	Highest
Ginger extract concentration (%) (*X*_*1*_)	40	60	80
SnCl_2_.2H_2_O concentration (M) (*X*_*2*_)	0.3	0.4	0.5
pH (*X*_*3*_)	5	6	7

**Table 2:** Randomized run orders of the design combinations and their responses

Std order	Run order	Ginger extract concentration (%) (*X*_*1*_)	SnCl_2_.2H_2_O concentration (M) (*X*_*2*_)	pH (*X*_*3*_)	Yield of SnO_2_ NPs (g)
19	1	80	0.5	6	0.1279
43	2	60	0.4	6	0.1026
36	3	80	0.4	5	0.1207
42	4	60	0.5	7	0.1276
44	5	60	0.4	6	0.1059
31	6	40	0.3	6	0.0824
32	7	80	0.3	6	0.1304
21	8	80	0.4	5	0.1986
39	9	60	0.3	5	0.1189
41	10	60	0.3	7	0.1274
15	11	60	0.4	6	0.2012
13	12	60	0.4	6	0.2142
14	13	60	0.4	6	0.2115
6	14	80	0.4	5	0.2277
12	15	60	0.5	7	0.1736
23	16	80	0.4	7	0.2298
2	17	80	0.3	6	0.1511
27	18	60	0.5	7	0.1818
25	19	60	0.5	5	0.3213
29	20	60	0.4	6	0.2910
11	21	60	0.3	7	0.1925
45	22	60	0.4	6	0.2871
1	23	40	0.3	6	0.1836
20	24	40	0.4	5	0.2875
30	25	60	0.4	6	0.2960
38	26	80	0.4	7	0.3235
5	27	40	0.4	5	0.2814
28	28	60	0.4	6	0.3219
24	29	60	0.3	5	0.2065
17	30	80	0.3	6	0.2321
22	31	40	0.4	7	0.2749
33	32	40	0.5	6	0.3154
4	33	80	0.5	6	0.3731
26	34	60	0.3	7	0.2140
37	35	40	0.4	7	0.2781
3	36	40	0.5	6	0.3407
9	37	60	0.3	5	0.2200
10	38	60	0.5	5	0.3524
16	39	40	0.3	6	0.2007
34	40	80	0.5	6	0.3666
35	41	40	0.4	5	0.2784
7	42	40	0.4	7	0.3025
40	43	60	0.5	5	0.3690
8	44	80	0.4	7	0.3123
18	45	40	0.5	6	0.3266

For better understanding, the independent variables were denoted  as *X*_*1*_, *X*_*2*_, and *X*_*3*_ for ginger extract concentration, SnCl_2_.2H_2_O concentration, and pH, respectively. The percentage of ginger extract was determined based on dilutions of the original extract (0.05 g mL^−1^), which was designated as 100%. Accordingly, concentrations of 0.04, 0.03, 0.02, and 0.01 g mL^−1^ correspond to 80%, 60%, 40%, and 20%, respectively.

From the regression analysis on the experimental data, the relationship between the response variable and the three independent variables was determined, as presented  by the following second-order polynomial equation (Eq. 5).

5 $$Y=1.106-\left(0.02555 {X}_{1}\right)+\left(2.48 {X}_{2}\right)-\left(0.233 {X}_{3}\right)+\left(0.000112 {X}_{1} {X}_{1}\right)+\left(0.41 {X}_{2} {X}_{2}\right)+\left(0.0278 {X}_{3} {X}_{3}\right)+\left(0.00992 {X}_{1} {X}_{2}\right)+\left(0.001293 {X}_{1} {X}_{3}\right)-\left(0.473 {X}_{2} {X}_{3}\right)$$

where *Y* is the yield of SnO_2_ NPs (g) and *X*_*1*_, *X*_*2*_, and *X*_*3*_ are the independent variables which are ginger extract concentration (%), SnCl_2_.2H_2_O concentration (M), and pH, respectively.

Statistical testing of the model was performed using analysis of variance (ANOVA). The coefficient of determination (*R*^2^ = 0.5276 or 52.76%) indicated  a moderate influence,  meaning that 47.24% of the variability in the outcome data was not explained by Eq. 5, whereas the coefficient of adjusted determination (adjusted *R*^2^ = 0.4062 or 40.62%) indicated a weak influence. This was due to the fact that , among  the 3 variables (*X*_*1*_ = ginger extract concentration (%), *X*_*2*_ = SnCl_2_.2H_2_O concentration (M), and *X*_*3*_ = pH), only *X*_*2*_ contributed  significant value to the model. Despite the relatively low adjusted *R*^2^ value, the model (Eq. 5) was considered significant based on the *F*-value and *P*-value, which were 4.34 and 0.001, respectively. These values suggest strong evidence for the overall significance of the model. The model's validity was further supported  by the model’s lack-of-fit test, which yielded *F*-values of 9.51 and *P*-values of 0.000, respectively. These results indicate a 0% probability  that non-random elements might influence the model’s prediction.

The significance of the three independent variables was assessed based on their *F*-values and *P*-values. The *F*-values for ginger extract concentration, SnCl_2_.2H_2_O concentration, and pH were 0.28, 19.78, and 0.77, respectively. Correspondingly, the *P*-values were 0.601, 0.000, and 0.385, respectively. According to the general criteria for statistical significance (*F* > 2.5 and *P* < 0.05), only the SnCl_2_.2H_2_O concentration showed both a significant F- and P-values, indicating its strong  influence on the yield of SnO_2_ NPs.

#### Relationship Between Independent Variables on SnO_2_ NPs Production

**Fig. 2:**
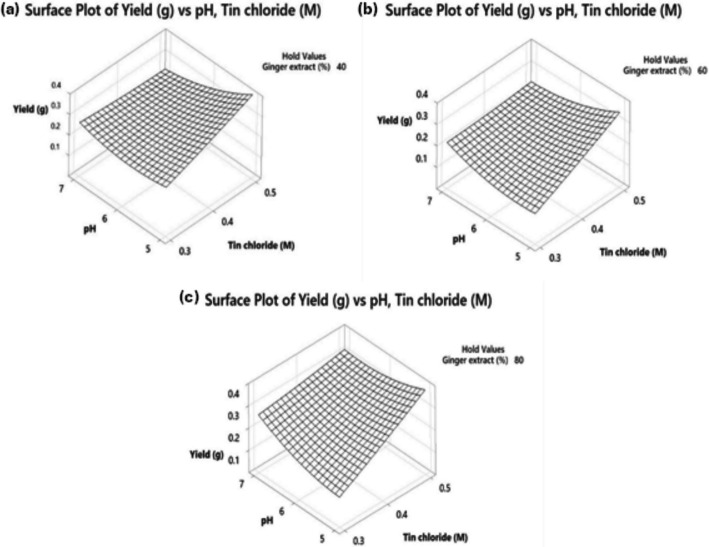
Surface plots showing the effects of SnCl_2_.2H_2_O concentration (M) and pH on the yield of SnO_2_ NPs when the ginger extract concentration (%) is kept constant at **a** 40%, **b** 60%, and **c** 80%

3D response surface plots were generated  to study the effects of the three independent variables on the yield of SnO_2_ NPs. From the statistical analysis, only SnCl_2_.2H_2_O concentration and pH of reaction showed a significant two-way interaction (*P*-value = 0.011) on SnO_2_ NPs production. Thus, the plots presented in Fig. 2a, b, and c focus on the synergistic effects of SnCl_2_.2H_2_O concentration and pH at fixed ginger extract concentration of 40, 60, and 80%, respectively. All three  plots show that the yield of SnO_2_ NPs increased when the SnCl_2_.2H_2_O concentration increased from 0.3 to 0.5 M and when the pH decreased from pH 7 to 5. However, the most  pronounced increase in the yield of SnO_2_ NPs occurred when the ginger extract concentration was fixed at 40% (Fig. 2a). Overall, the best yield of SnO_2_ NPs was obtained when the ginger extract concentration, SnCl_2_.2H_2_O concentration, and pH were at 40% (0.02 g mL^−1^), 0.5 M, and 5, respectively. These values were considered  the optimum condition for the photocatalyst synthesis, with a constant reaction time and temperature of 1 h and 65 °C, respectively.

#### Comparison Between the Theoretical and Experimental Yields of SnO_2_ NPs

The mathematical model shown in Eq. 5 was verified by testing the yield of SnO_2_ NPs using the optimized and a random combination  of the three variables, as shown in Table 3. The total volume of the solution (mixture of ginger extract and SnCl_2_.2H_2_O) used to synthesize SnO_2_ NPs for each batch  was fixed at 25 mL (20 mL of ginger extract + 5 mL of SnCl_2_.2H_2_O solution). The SnO_2_ NPs yields (obtained experimentally) for both batches were compared with the theoretical yields (calculated using Eq. 5). The differences in mass between the experimental and theoretical yields of SnO_2_ NPs for the optimized  and random batches were 0.0371 and 0.0081 g, respectively. The percent errors (calculated using Eq. 2) for both the batches were 9.229 and 3.635%, respectively- both  within acceptable range (≤ 10%). This shows that the generated Eq. 5 reliably predicts the yield of SnO_2_ NPs, indicating the model's accuracy and applicability.

**Table 3:** The percentage differences (percent errors, %) of the theoretical and experimental yields of SnO_2_ NPs for optimal and random batches

Batch	Factors			Total volume (mL)	Experimental yield of SnO_2_ NPs (g)	Theoretical yield of SnO_2_ NPs (g)	Percentage difference (%)
	*X*_*1*_	*X*_*2*_	*X*_*3*_				
Optimal	40%	0.5 M	5	25	0.3731	0.41020	9.229
Random	60%	0.4 M	7	25	0.2298	0.22174	3.635

### Characterization of SnO_2_ NPs

#### Evaluation of Crystalline Nature and Composition of SnO_2_ NPs Using Powder X-ray Diffraction (XRD) Analysis

**Fig. 3:**
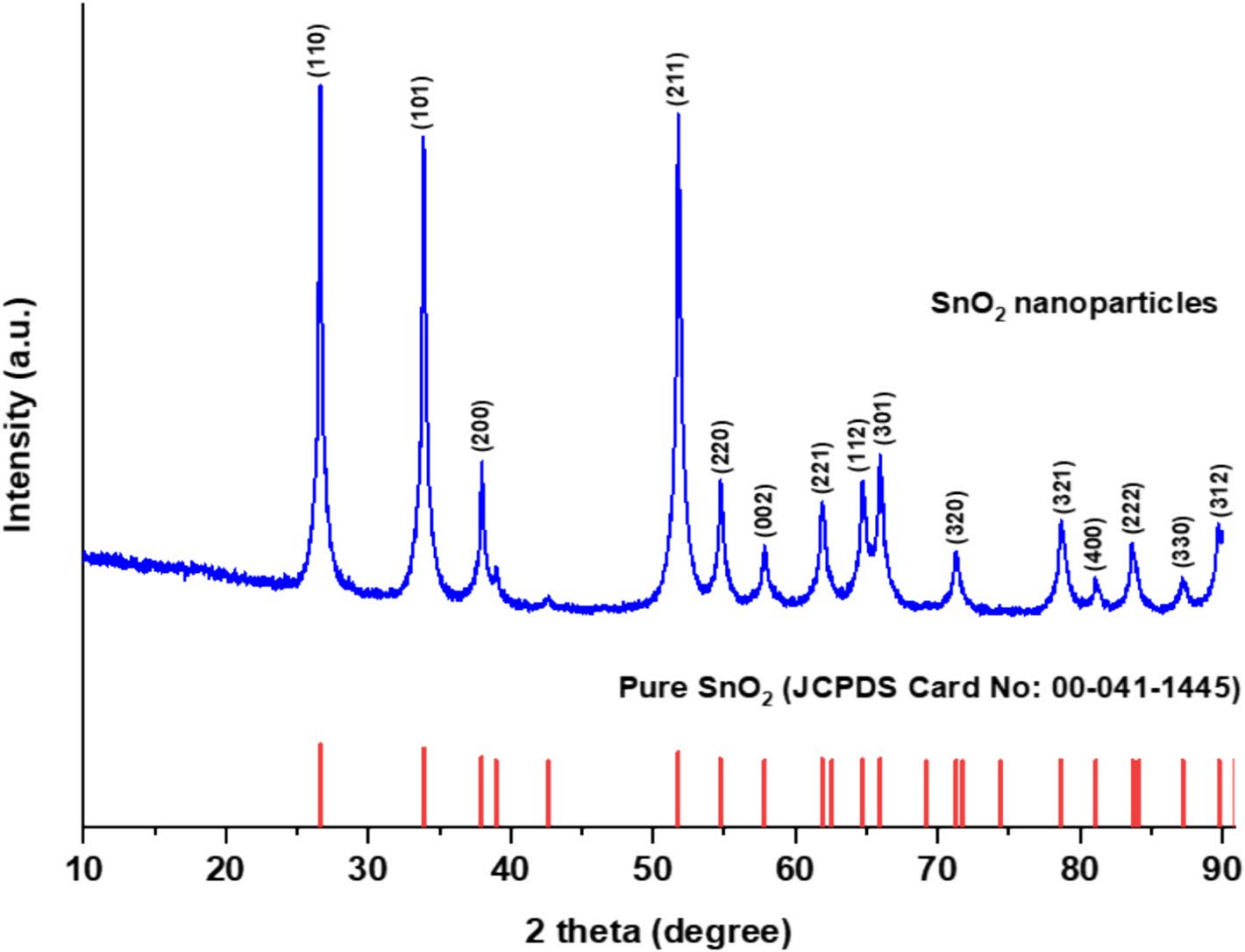
X-ray diffraction (XRD) spectrum of SnO_2_ NPs sample

XRD pattern of SnO_2_ NPs is shown in Fig. 3 and was used to determine the crystal structure (crystallinity) and composition of SnO_2_ NPs. The diffraction pattern of SnO_2_ NPs exhibited  reflections at 2 theta, θ = 26.63°, 33.93°, 38.07°, 51.85°, 54.74°, 57.77°, 62.32°, 64.66°, 65.91°, 71.82°, 78.72°, 81.20°, 84.08°, 87.25°, and 89.88° which correlated with the diffraction peak *hkl* values of lattice planes = (110), (101), (200), (211), (220), (002), (221), (112), (301), (320), (321), (400), (222), (330), and (312), respectively. All the diffraction peaks matched well with the reported Joint Committee Powder Diffraction Standards, JCPDS = 00–041–1445, which is associated with the tetragonal structure of SnO_2_. The sharp and narrow diffraction peaks indicated that the particles have good crystallinity and uniform size. Furthermore , the XRD pattern shows only monophasic diffraction with no detectable impurity peaks, confirming the high purity of the synthesized SnO_2_ NPs photocatalyst. The crystallite size of SnO_2_ NPs can be calculated using Scherrer equation (Eq. 6), as shown below.

6 $$D = K\lambda / \beta cos(\theta )$$

where $D$ is the crystallite size (nm), $K$ is the Scherrer constant (0.9), $\lambda$ is the X-ray wavelength (0.15406 nm), $\beta$ is the full width at half maximum (FWHM) (radians), and $\theta$ is the Bragg angle (peak position).

The average crystallite size of the synthesized SnO_2_ NPs calculated using Eq. 6 was determined to be  23.7064 nm. This confirms  that the SnO_2_ NPs are indeed in the nanocrystalline range,as the size falls  within the typical nanoparticle diameter of 1 to 100 nm. Based on previous studies on SnO₂ NPs, a similar crystallite size of approximately 23 nm was also reported  [93]. _2_

#### Identification of Functional Groups on SnO_2_ NPs Surface Using Fourier Transform Infrared (FTIR) Analysis

**Fig. 4:**
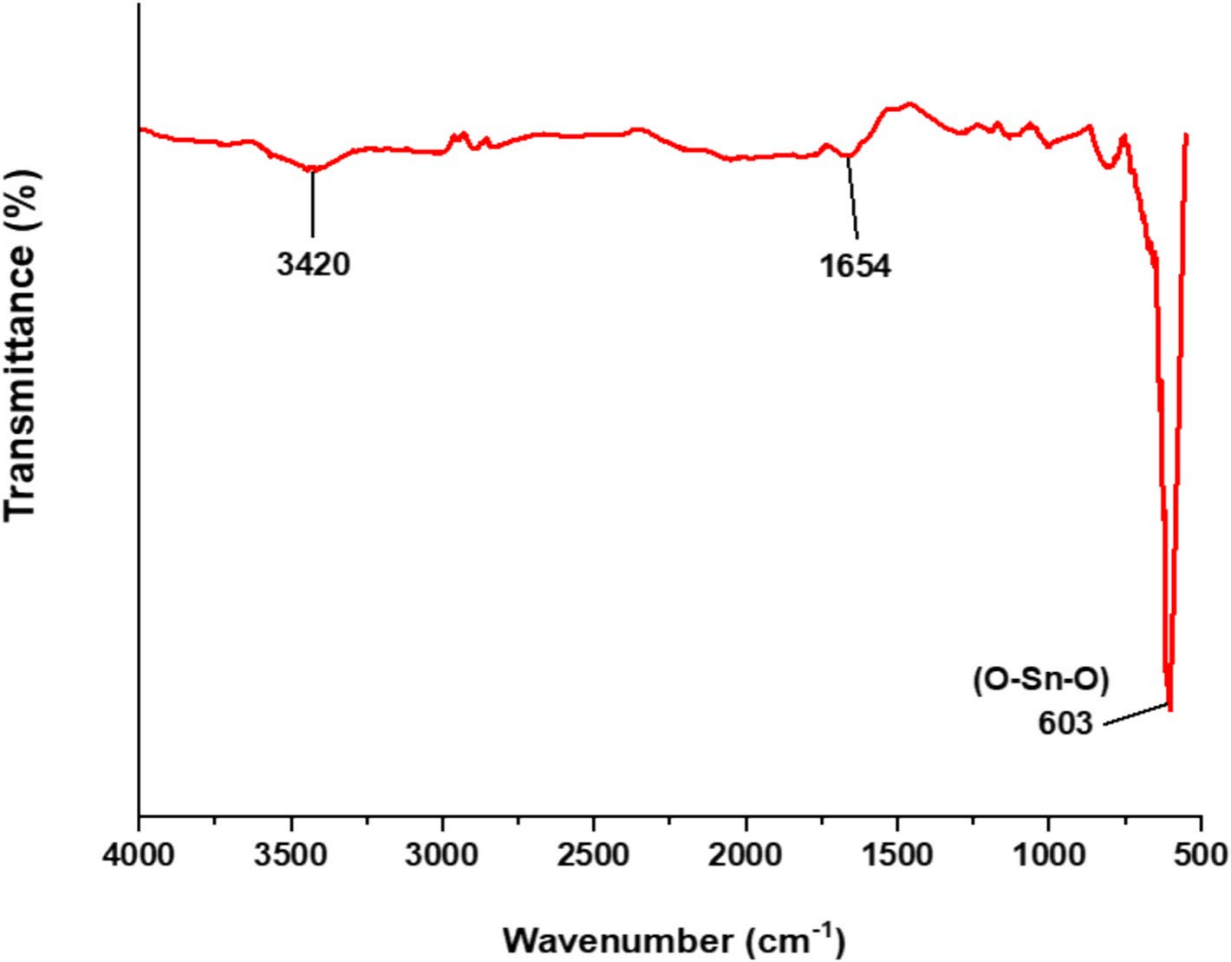
Fourier transform infrared (FTIR) spectrum of SnO_2_ NPs sample

FTIR analysis was conducted to verify  the bond structure and  the  functional groups present in  the synthesized SnO_2_ NPs. The infrared absorption spectrum of the SnO_2_ NPs shown in Fig. 4 was recorded in the wavenumber range of 4000–500 cm^−1^. The absorption bands at 3420 cm^−1^ [63], 1654 cm^−1^ [86], and 603 cm^−1^ [56] can be attributed  to stretching vibrations of the hydroxyl (-OH), alkene (-C = C-), and oxygen-related (O–Sn–O) groups, respectively. This proved that the SnO_2_ NPs sample contains stretching and bending biomolecule vibrations on their surface. Similar findings were reported in a previous study [70], where absorption of SnO_2_ NPs was observed in the range of 540–660 cm^−1^.

#### Ultraviolet–Visible (UV–Vis) Analysis

**Fig. 5:**
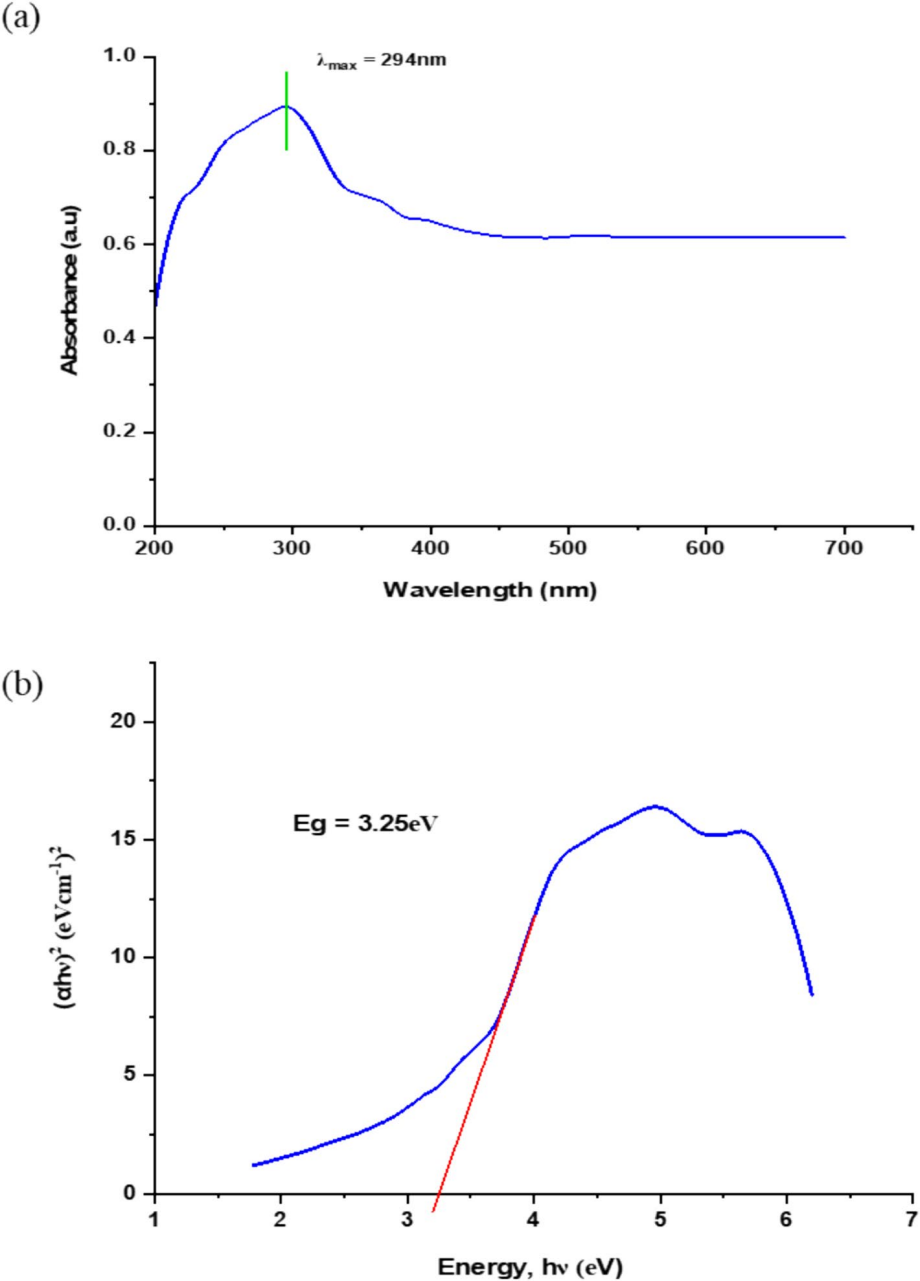
**a** UV–Vis absorption spectrum and **b** band gap energy of SnO_2_ NPs sample

UV–Vis analysis was performed to measure the SnO_2_ NPs full-wavelength absorbance and to confirm their potential for photocatalytic activity. Figure 5a shows  the UV–Vis absorption spectrum characteristic of SnO_2_ NPs in the wavelength range of 200–700 nm, with a prominent  absorption band at *λ*_max_ = 294 nm. Based on this absorption spectrum (Fig. 5a), the band gap energy (Eg), which is an optical property that expresses the semiconducting nature and the absorption coefficient of SnO_2_ NPs, was determined using the Tauc plot method (Eq. 7), as shown below.

7 $$\left(\alpha hv\right)n = A\left(hv-Eg\right)$$

where $\alpha$ is the absorption coefficient, $hv$ is the photon energy, $n$ is the nature of transmission, $A$ is the energy independent constant, and $Eg$ is the optical band gap.

The band gap energy of SnO_2_ NPs was determined  to be 3.25 eV (Fig. 5b), which is consistent with the value reported in previous studies [32, 84], where the band gap ranged from  3.1 and 3.9 eV [7]. This band gap energy enables  the excitation of electrons from the valence band to the conduction band under ultraviolet light irradiation (λ = 294 nm), thereby facilitating the photocatalytic effect.

###  Photocatalytic Activity of SnO_2_ NPs

**Fig. 6:**
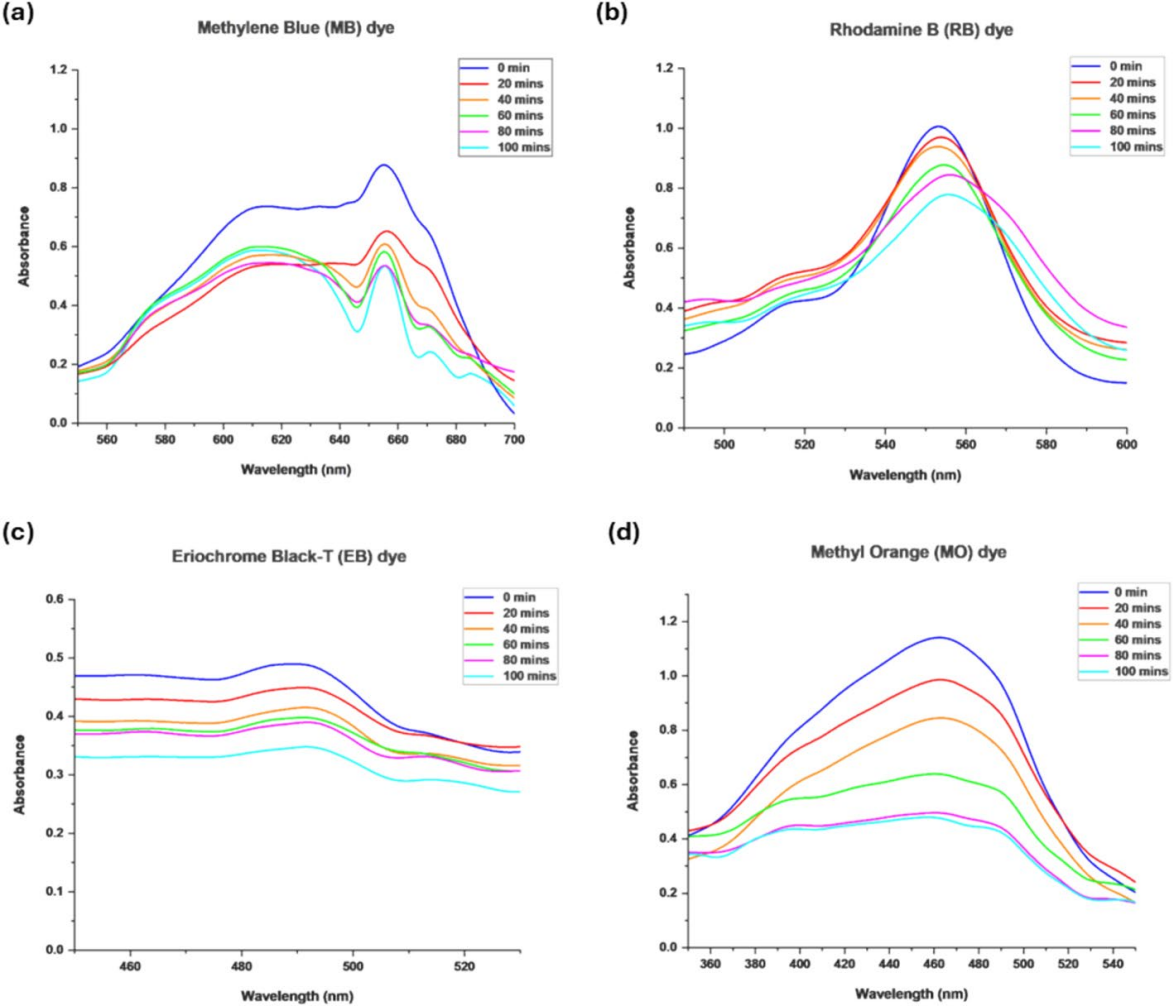
UV absorbance of **a** MB, **b** RB, **c** EB, and **d** MO dyes upon photodegradation by SnO_2_ NPs at the intervals of 20 min for 100 min

SnO_2_ NPs was tested for photodegradation of four different dyes: methylene blue (MB), rhodamine B (RB), Eriochrome black-T (EB), and methyl orange (MO). The concentration of RB dye (5 ppm) used in this experiment differed  from the other dyes (10 ppm), as it was selected based on the preliminary optimization experiment. At a concentration of 10 ppm, the maximum absorbance of RB dye exceeded the optimal range for accurate spectrophotometric measurement. By reducing the concentration to 5 ppm, the absorbance fell within a measurable range, allowing the photocatalytic efficiency to be reliably assessed based on the observed decrease in RB dye concentration. Hence, this allows comparisons to be made for the dyes tested. The photocatalytic degradation of these dyes by SnO_2_ NPs was carried out under UV light (*λ*_max_ = 294 nm) irradiation. Figure 6 shows the absorbance spectra of the dyes during photodegradation, recorded at 20-min intervals over a 100-min period in the range of 300–700 nm. From the results, all dyes undergo significant degradation catalyzed by SnO_2_ NPs, indicating its good photocatalytic activity. This is supported by other studies whereby the tetragonal structure of SnO_2_ NPs showed high photocatalytic activity and viability in the removal of dyes [66, 68]. The proposed mechanism of dyes degradation in the presence of SnO_2_ NPs under irradiation of UV light involves several steps of reaction succession. Upon UV exposure, electrons are excited  and transferred from the valence band to the conduction band with corresponding energy higher than SnO_2_ NPs band gap energy. This will promote holes in the valence band as well as generate electrons in the conduction band. This process generates high reactive oxygen species (ROS) like hydroxyl radicals which are reactive with MB, RB, EB, and MO dye molecules [66]. Details of the mechanism are described in Supplementary Figure S5.

**Fig. 7:**
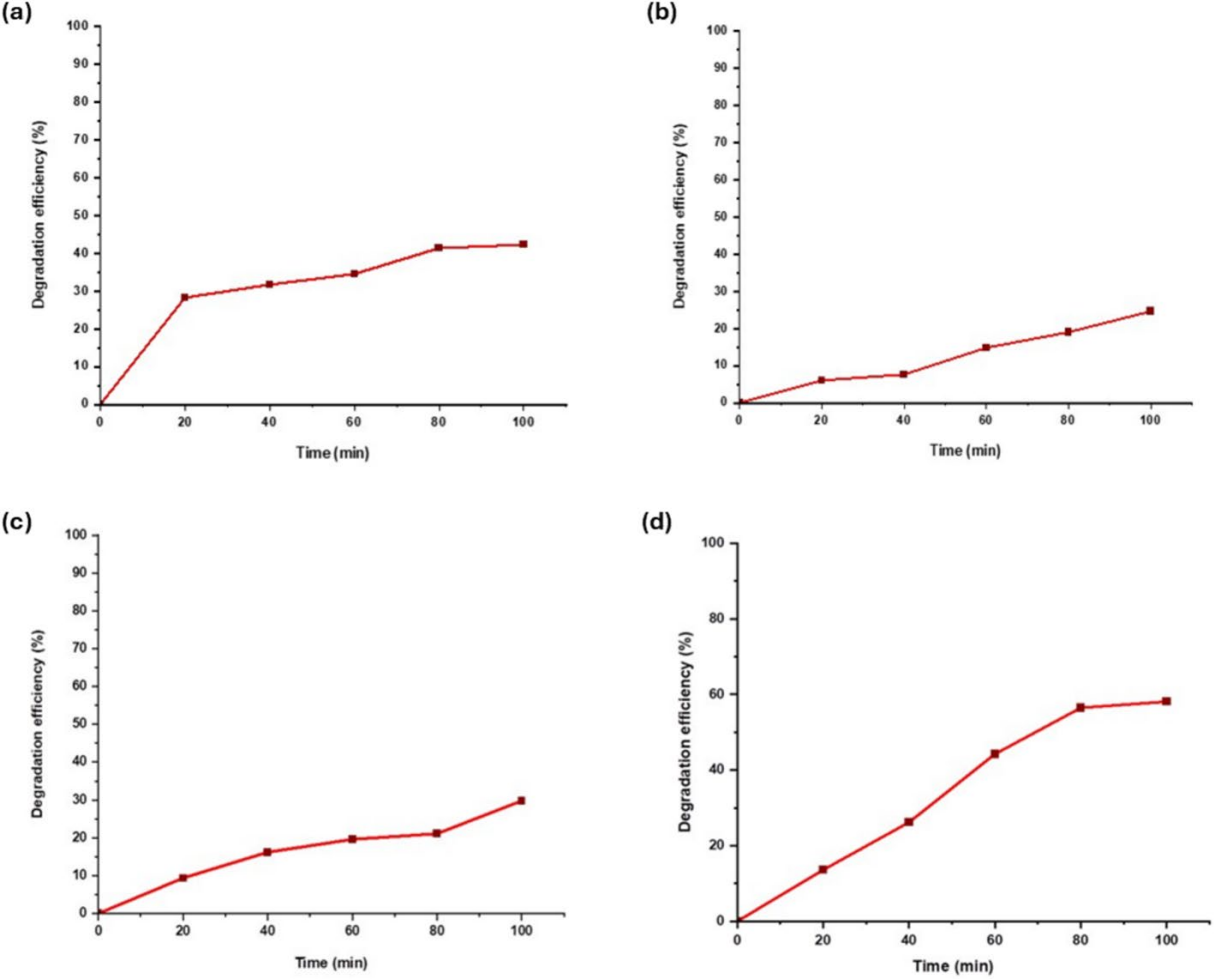
Photocatalytic efficiency (%) of SnO_2_ NPs in degrading **a** MB, **b** RB, **c** EB, and **d** MO dyes

The degradation efficiency of SnO_2_ NPs for all dyes was then calculated using Eq. 3. Based on Fig. 7a, the MB dye degradation efficiency was found to be 42.25% during the duration of 100 min. It shows an increase in the degradation in every 20-min interval, suggesting that the degradation efficiency would be above 95% with the addition of another 100 min (total of 200 min). This result is supported by a previous study [36] whereby the degradation efficiency of MB is reported to be 85% in 3 h (180 min) using ZnO nanoparticles under UV radiation. The RB dye degradation efficiency is shown to be 24.62% during the duration of 100 min (Fig. 7b). From the graph trend, the degradation efficiency would be 95% over another 300 min. A similar result was reported by a study [17] whereby the degradation efficiency of RB is 69.23% in 4 h (240 min) using CuS nanoparticles under UV radiation. For EB degradation, the efficiency was 29.78% during the duration of 100 min (Fig. 7c). It is expected that the degradation efficiency would reach 95% when the process is prolonged for another 260 min. On the other hand, the degradation efficiency of MO was the highest (58.10%) among the other tested dyes. The MO was degraded much faster with the expectation of being 95% degraded within 180 min (Fig. 7d). In a previous study [67], the degradation efficiency of MO is 90% in 3 h (180 min) using ZnO nanocatalysts under UV radiation, thus supporting the present result. Overall, data analysis demonstrated that the synthesized SnO_2_ NPs using ginger extract exhibit promising photocatalytic performance in the degradation of all dyes in this study. The variation in the degradation efficiency of each dye is likely attributed to the disparity in their molecular structure, with MO having the simplest structure, thus making it more susceptible to fast degradation.

The kinetics plots of photocatalytic degradation of  MB, RB, EB, and MO dyes using SnO_2_ NPs, are depicted in Supplementary Figure S3. They show that the degradation of the dyes follows pseudo-first-order kinetics. Based on Supplementary Figure S4, the half-life of the dyes was determined through the intersection point between the degradation efficiency curve (1 – *C*_*t*_/*C*_*o*_) and the dye concentration curve (*C*_*t*_/*C*_*o*_). The calculated half-life for MB, RB, EB, and MO dyes were 16.38, 53.56, 45.64, and 44.21 min, respectively. These values represent the time required for the initial concentrations of the dyes to reduce by half.

These results demonstrate that SnO_2_ NPs exhibit significant photocatalytic activity and can be effectively employed in wastewater treatment, thereby contributing to environmental remediation. This efficacy arises from the SnO_2_ NPs’ ability to be activated by both UV and visible light, as well as their good charge separation properties and high structural stability. However, the efficiency of SnO_2_ NPs in degrading pollutants like dyes is influenced by several factors, including pollutant concentration, light intensity, pH, and temperature. The rate of degradation is generally directly proportional to the pollutant concentration, while pH impacts both the reactivity and adsorption of pollutants on the surface of SnO_2_ NPs, thus affecting the overall degradation efficiency. Increased light intensity typically enhances the degradation rate; however, at excessively high intensities, saturation effects may occur, limiting further improvement. Temperature also plays a crucial role in influencing the kinetics of the photocatalytic reaction, with higher temperatures generally accelerating the rate of reaction.

### Toxicity Effect of SnO_2_ NPs on Brine Shrimp (*Artemia salina*)

The mortality results of *Artemia salina* exposed to varying concentrations of SnO_2_ NPs (sample) and K_2_Cr_2_O_7_ (standard) over 24 h are shown in Tables 4 and 5, respectively. K_2_Cr_2_O_7_ was used as the standard in this toxicity test because it is a common inorganic chemical reagent that is used as a reference toxicant in aquatic toxicity testing [49]. It is toxic to aquatic organisms, soluble in water, a strong oxidizing agent, and non-deliquescent.

**Table 4:** Percentage of *Artemia salina* killed under different concentrations, ranging from 1.56 to 400 µg mL^−1^ of SnO_2_ NPs in 24 h exposure time

Concentration (µg mL^−1^)	Initial number of nauplii	Mean of living nauplii after 24 h	Percentage of mortality of *Artemia salina* (%)
0 (control)	10	10	0
1.56	10	10	0
10	10	10	0
50	10	9.667	3.33
100	10	9.333	6.67
250	10	9.333	6.67
400	10	6.667	33.33

**Table 5:** Toxicity testing of standard, K_2_Cr_2_O_7_ on *Artemia salina* and percentage of *Artemia salina* killed under different concentrations, ranging from 1 to 100 µg mL^−1^ of standard in 24 h exposure time

Concentration (µg mL^−1^)	Initial number of nauplii	Mean of living nauplii after 24 h	Percentage of mortality of *Artemia salina* (%)
0 (control)	10	10	0
1	10	7.333	26.67
10	10	7	30
25	10	5.333	46.67
50	10	0	100
75	10	0	100
100	10	0	100

Based on Table 4, it can be deduced that SnO_2_ NPs exhibit no toxicity within the concentration range of 1.56–10 µg mL^−1^. At concentrations between 50 and 250 µg mL^−1^, the toxicity is negligible, with mortality rates of only of 3.33% at 50 µg mL^−1^ and 6.67% at concentrations ranging from 100 to 250 µg mL^−1^. In general, SnO_2_ NPs is considered safe to be used in aquatic environments, as the survival rate of *Artemia salina* remained above 90% across concentration range of 1.56–250 µg mL^−1^. Noticeable toxicity was only observed when the concentration of SnO_2_ NPs was increased to 400 µg mL^−1^, where mortality of *Artemia salina* exceeded 10%. However, SnO_2_ NPs are still deemed safe for aquatic environment with concentration below this threshold. In this study, only 0.02 g of SnO_2_ NPs was used to degrade 60 mL (5 ppm and 10 ppm) of dyes, which is equivalent to 5 µg mL^−1^ and 10 µg mL^−1^, respectively. At this concentration, no mortality was observed in *Artemia salina*, well below the level at which toxicity begins to manifest. Comparing Tables 4 and 5, SnO_2_ NPs shows lower toxicity on *Artemia salina* (< 10% at concentration range of 1.56–250 µg mL^−1^ and < 50% at 400 µg mL^−1^) compared to the standard, K_2_Cr_2_O_7_, which is more than 10% at all the concentrations. The mortality of *Artemia salina* reaches 100% when the concentration of K_2_Cr_2_O_7_ reaches 50 µg mL^−1^. This finding highlights that SnO_2_ NPs is considered to be non-toxic and safe for use in aquatic environments within the concentrations range of 1.56–250 µg mL^−1^ as compared to the standard K_2_Cr_2_O_7_.

**Fig. 8:**
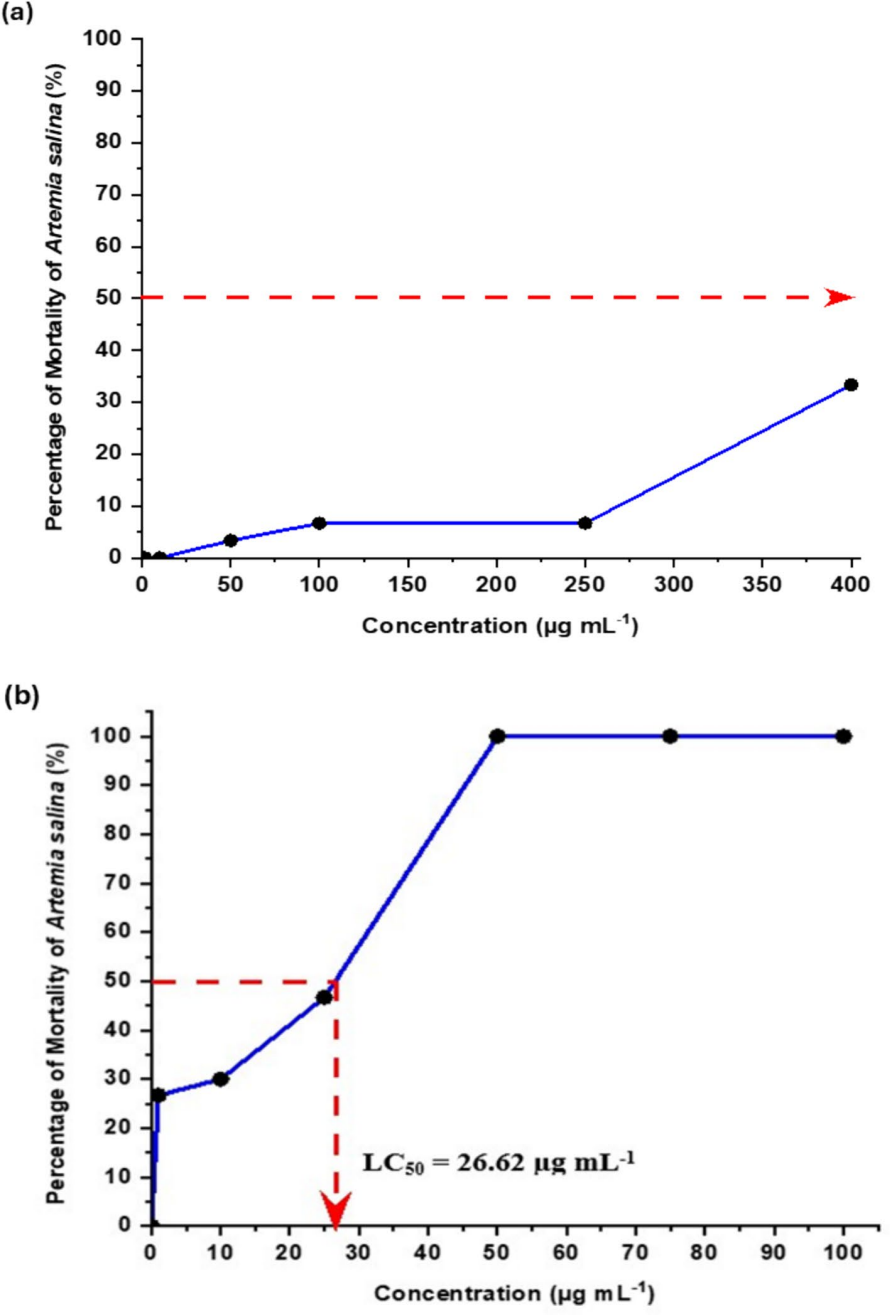
Relationship between **a** SnO_2_ NPs concentrations ranging from 0 to 400 µg mL^−1^ and **b** standard, K_2_Cr_2_O_7_ concentrations ranging from 0 to 100 µg mL.^−1^ with the percentage of mortality of *Artemia salina* in 24 h exposure time. Red arrows represent LC_50_ value for SnO_2_ NPs and K_2_Cr_2_O_7_

The graphs plotted in Fig. 8 presented below show the relationship between the concentrations and percentage of mortality of *Artemia salina* for both SnO_2_ NPs (Fig. 8a) and K_2_Cr_2_O_7_ (Fig. 8b) in 24 h of exposure time. The results clearly show that the mortality percentage of *Artemia salina* increased with the increase in concentrations of both SnO_2_ NPs and K_2_Cr_2_O_7_. Thus, this indicates a direct, proportional relationship between concentration and mortality, meaning that as the concentration increases, mortality also increases, and vice versa.

The lethal concentrations (LC₅₀) of SnO₂ NPs and the standard, K₂Cr₂O₇, towards *Artemia salina* were determined in this test, as presented by red arrows in Fig. 8. As shown in Fig. 8a, SnO₂ NPs did not exhibit an LC₅₀ value (the concentration at which 50% mortality of *Artemia salina* occurs) after 24 h. In contrast, Fig. 8b shows that the LC₅₀ of K₂Cr₂O₇ is 26.62 µg mL^−1^, indicating greater toxicity compared to SnO₂ NPs. Therefore, K₂Cr₂O₇ can be safely used in aqueous environments as long as its concentration does not exceed 26.62 µg mL^−1^. On the other hand, since no LC₅₀ value was observed for SnO₂ NPs, with no concentration causing 50% mortality of *Artemia salina*, the exact “safe” concentration for SnO₂ NPs cannot be precisely determined from this test. However, based on Fig. 8a, the concentrations tested (ranging from 0 to 400 µg mL^−1^) are safe for use in aqueous environments for dye photocatalysis. It can be concluded that the LC₅₀ for SnO₂ NPs is above 400 µg mL^−1^.

## Conclusions

A sustainable and non-toxic approach for synthesizing tin oxide nanoparticles (SnO_2_ NPs) using ginger extract has proven to be successful. The optimal synthesis conditions were found to be a ginger extract concentration of 0.02 g mL^−1^, 0.5 M of tin (II) chloride dihydrate (SnCl_2_·H_2_O), and a pH of 5. The resulting SnO_2_ NPs exhibited a band gap of 3.25 eV, which lies within the ideal range (3.1–3.9 eV) for effective photon activation, thereby demonstrating strong photocatalytic degradation potential for various dyes. Specifically, the degradation efficiencies of methylene blue (MB), rhodamine B (RB), Eriochrome black-T (EB), and methyl orange (MO) were 42.25%, 24.62%, 29.78%, and 58.10%, respectively, after 100 min. A toxicity assessment of SnO_2_ NPs was conducted using brine shrimp (*Artemia salina*), confirming their safety in aquatic environments, even at concentrations effective for dye degradation, which are significant pollutants in water systems. The mortality rate of *Artemia salina* remained below 10% across a concentration range of 1.56–250 µg mL^−1^. Notably, the optimum concentration of SnO_2_ NPs for dye degradation was determined to be 10 ppm (10 µg mL^−1^), which resulted in 0% mortality for *Artemia salina*. These findings collectively highlight that SnO_2_ NPs synthesized with ginger extract represent a promising, environmentally friendly photocatalyst for dye degradation, offering a sustainable solution for mitigating water pollution.

## Supplementary Information

Below is the link to the electronic supplementary material.

Supplementary file1 (PDF 400 KB)

## Data Availability

Not applicable.
