# Supplementary material for: Green Synthesis of Tin Oxide (SnO2) Nanoparticles Using Ginger Extracts for Photocatalytic Degradation of Organic Dyes in Wastewater
Source: Appl Biochem Biotechnol. 2025 Jun 27;197(9):5668–93. doi: 10.1007/s12010-025-05293-2 (PMC12568811; doi:10.1007/s12010-025-05293-2)
Supplement: Supplementary file 1 — Supplementary file1 (PDF 400 KB) [file 12010_2025_5293_MOESM1_ESM.pdf]

Supplementary Information

Green Synthesis of Tin Oxide (SnO_2) Nanoparticles using Ginger Extracts for Photocatalytic Degradation of Organic Dyes in Wastewater

Yuvana Sivarajan[1], Khairul Anwar Ishak[2] and Jamilah Syafawati Yaacob[1,3*]

[1] Institute of Biological Sciences, Faculty of Science, Universiti Malaya, 50603 Kuala Lumpur, Malaysia.

[2] Institute of Systems Biology, Universiti Kebangsaan Malaysia, 43600 UKM Bangi Selangor, Malaysia

[3] Centre for Research in Biotechnology for Agriculture (CEBAR), Institute of Biological Sciences, Faculty of Science, Universiti Malaya, 50603 Kuala Lumpur, Malaysia.

*Correspondence: jamilahsyafawati@um.edu.my; Tel: +603-79674090

1. TFC Determination of Ginger Extract

The total flavonoid content (TFC) of 20 mg mL^{-1} (optimum concentration to synthesize SnO_2 NPs) ginger extract was calculated using quercetin as standard. The standard curve of quercetin was plotted, and used to determine the TFC of the ginger extract (based on Equation 1). The TFC of the ginger extract was 27.297 mgQE/g dry extract, and the high amount signifies high presence of flavonoids in the components of ginger extract. The standard plot for quercetin is shown below in Figure S1.

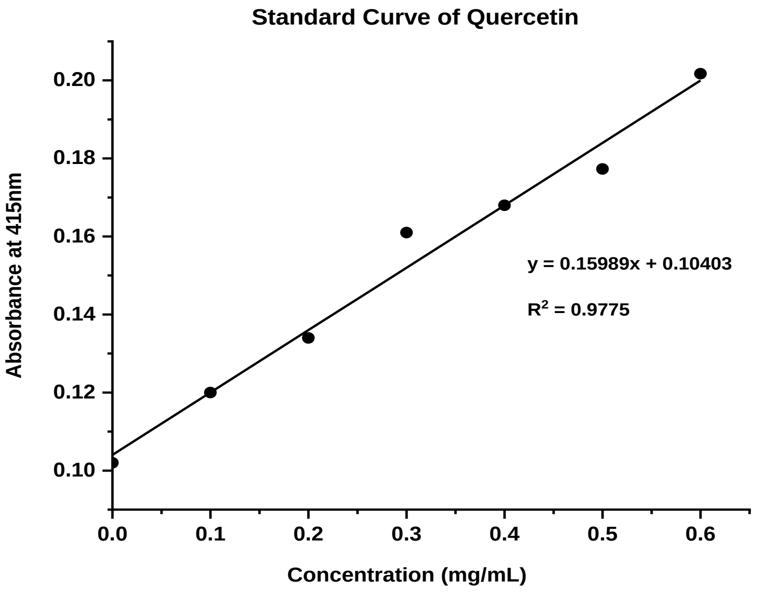

Supplementary Figure S1. Standard plot of Quercetin

2. Photocatalytic Activity of SnO_2 NPs on MB, RB, EB and MO Dyes

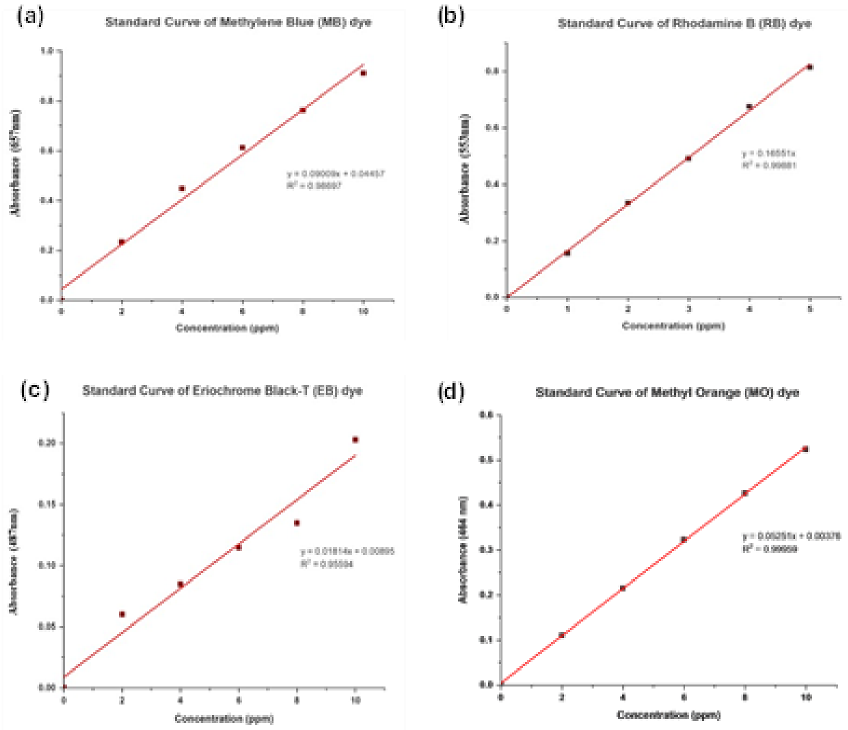

Supplementary Figure S2. Standard curve of (a) MB, (b) RB, (c) EB and (d) MO dyes

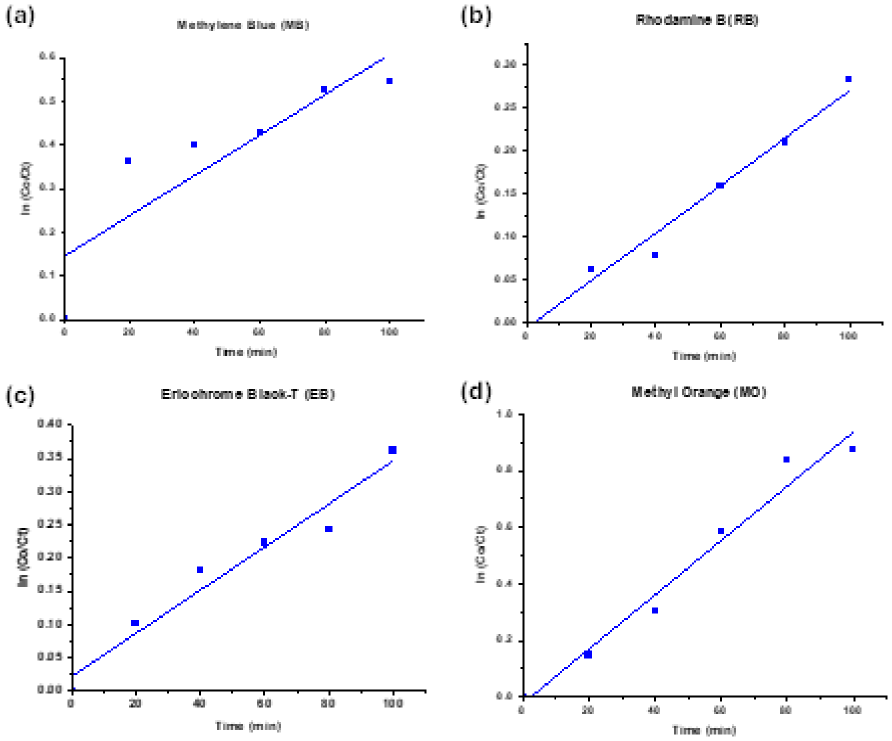

Supplementary Figure S3. Kinetic plots, ln (Co/Ct) vs. time (min) for photodegradation of (a) MB, (b) RB, (c) EB and (d) MO dyes.

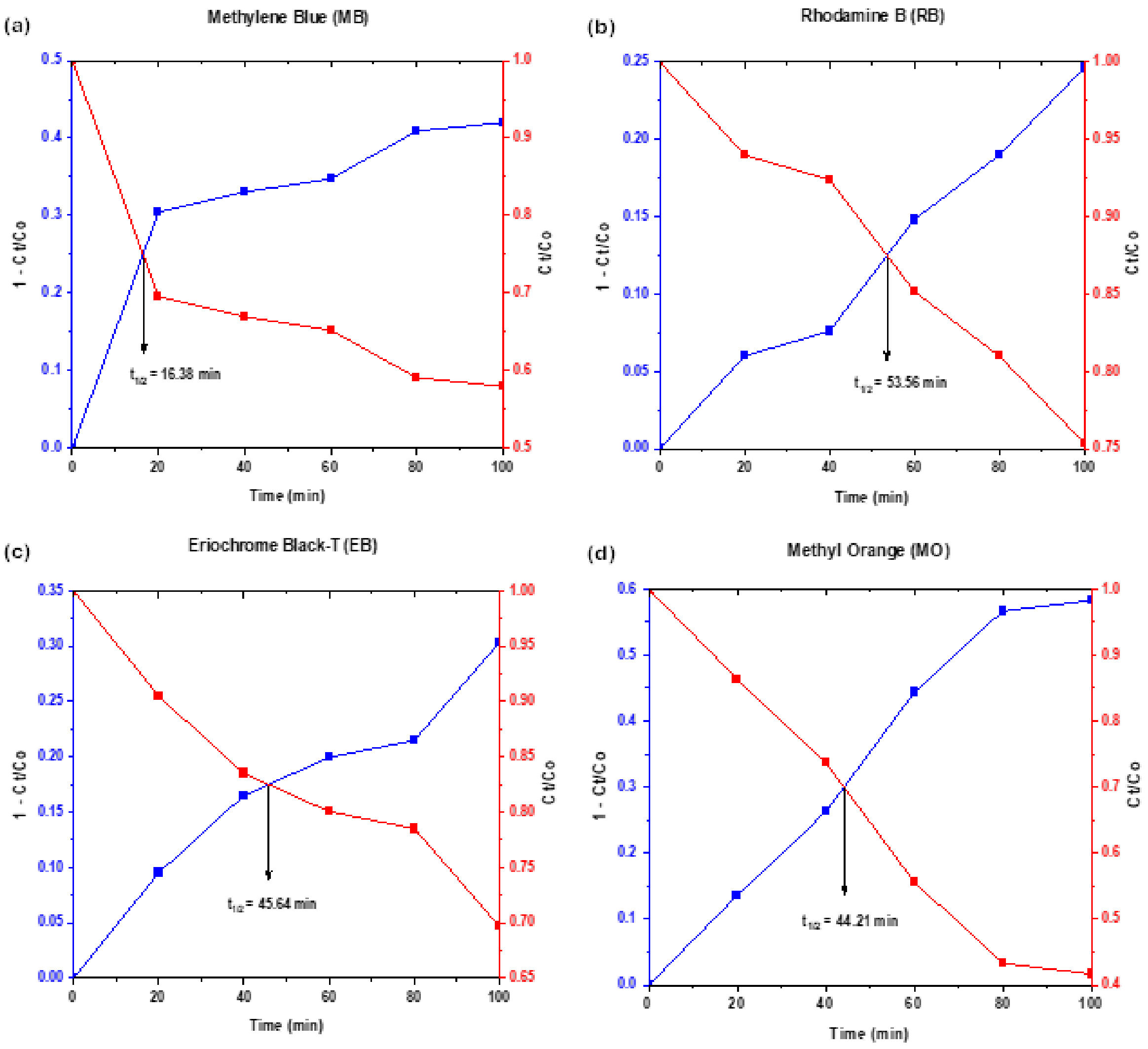

Supplementary Figure S4. Half-life estimation for degradation of (a) MB, (b) RB, (c) EB and (d) MO dyes.

3. Photodegradation Mechanism of MB, RB, EB and MO Dyes using Green Synthesized SnO_2 NPs

The cogent and feasible photodegradation mechanism of MB, RB, EB and MO dyes by the semiconductor SnO_2 NPs, synthesized in this research is exemplified below in Scheme S1 and Figure S5.

Scheme S1. Feasible Photodegradation Mechanism of MB, RB, EB and MO dyes by SnO_2 NPs.

$$SnO_2 \xrightarrow{h\nu} e^- + h^+ \quad (1)$$

$$e^- + O_2 \longrightarrow \dot{O}_2^- \quad (2)$$

$$O_2^- + H^+ \longrightarrow O\dot{O}H \quad (3)$$

$$H_2O + h^+ \longrightarrow O\bar{H} + H^+ \quad (4)$$

$$O\bar{H} + h^+ \longrightarrow \dot{O}H \quad (5)$$

$$\text{Dye [MB, RB, EB, MO]} \xrightarrow{h\nu} \dot{\text{Dye}} \quad (6)$$

$$\dot{\text{Dye}} + SnO_2 \longrightarrow \text{Dye} + SnO_2\,(e^-) \quad (7)$$

$$SnO_2\,(e^-) + O_2 \longrightarrow SnO_2 + O_2^- \quad (8)$$

$$SnO_2\,(e^-) + H_2O_2 \longrightarrow \dot{O}H + OH^- + SnO_2 \quad (9)$$

$$SnO_2 + (O\dot{O}H, \dot{O}_2^-) \longrightarrow \text{Intermediate products} \quad (10)$$

$$\downarrow$$

$$\text{Degraded products } (CO_2, H_2O) \quad (11)$$

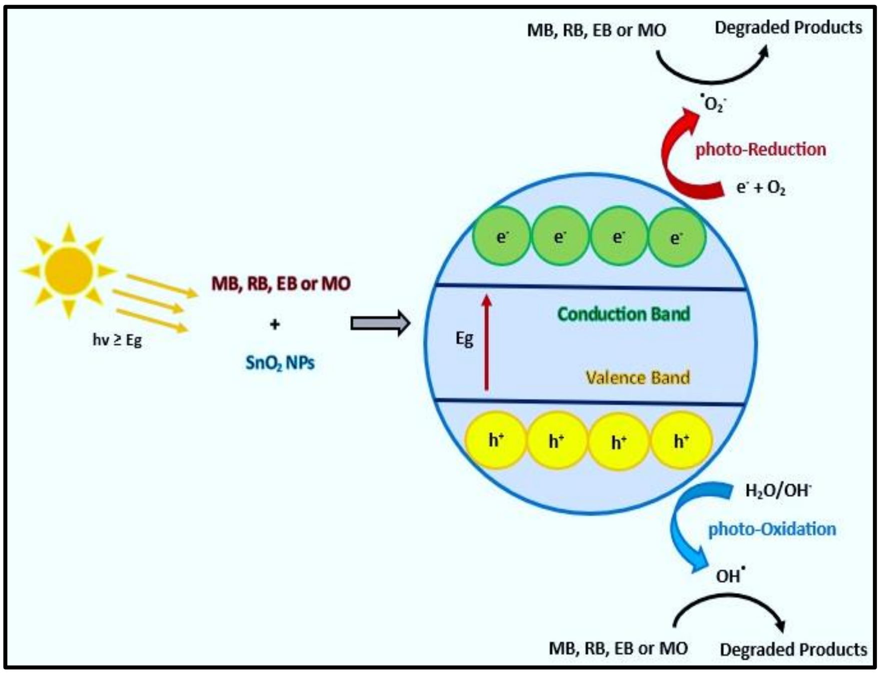

Supplementary Figure S5. MB, RB, EB and MO dyes photocatalytic degradation mechanism using SnO_2 NPs.

The possible mechanisms presented in Scheme S1 and Figure S5, describe several steps of reaction succession which occur during MB, RB, EB and MO dyes degradation in the presence of SnO_2 NPs under irradiation of UV light, assuming excess of highly reactive oxygen species (ROS) in the solution. A similar mechanism, but using ZnO NPs (Vidya et al., 2017) for photodegradation of dye in the presence of UV light is proposed.

Electrons move from the valence band to the conduction band in the presence of SnO_2 NPs under irradiation of UV light (equation 1). Then, conduction band electrons (e_{CB}^-) easily react with oxygen in order to produce superoxide ions (O_2^-), as shown in equation 2. Eventually, hydrogen ions (H^+) react with these highly reactive oxide ions so that -OOH is released, as shown in equation 3. Meanwhile, the holes easily get contact with water molecules in valence band ($h\nu_{VB}^+$) to generate hydroxide ions (OH^-), which successively produce highly active hydroxide radicals (•OH), as shown in equations 4 as well as 5. The generated -OOH can easily react with the conduction band electrons and hydrogen ions to generate highly active superoxide molecules (O_2^-). Ultimately, the cleavage of hydrogen peroxide molecules (H_2O_2) occurs in order to generate highly active hydroxyl radicals (•OH). The oxygen molecules (O_2) adsorbed on the surface of SnO_2 NPs catalyst are diminished by the photo-electrons to produce superoxide radicals ($O_2{\bullet}^-$). At the end, as represented by equations 6 to 11, the formed highly active hydroxyl (•OH) and superoxide radicals ($O_2{\bullet}^-$) decompose MB, RB, EB and MO dye molecules to generate mineral acids, carbon dioxide (CO_2) and water (H_2O).

4. Toxicity Effect of SnO_2 NPs in Comparison with Standard, $K_2Cr_2O_7$ on *Artemia salina*

Supplementary Table S1. Dilution series of SnO_2 NPs solution of different concentrations, ranging 0 – 400 µg mL^{-1} (0, 1.56, 10, 50, 100, 250 and 400 µg mL^{-1})

Concentration (µg mL^{-1})	Volume of Saltwater (µL)	Volume of SnO_2 NPs Stock Solution (µL)	Total Volume (µL)
0 (Control)	5000	0	5000
1.56	4984.4	15.6	5000
10	4900	100	5000
50	4500	500	5000
100	4000	1000	5000
250	2500	2500	5000
400	1000	4000	5000

Supplementary Table S2. Dilution series of $K_2Cr_2O_7$ standard solution of different concentrations, ranging 0 – 100 µg mL^{-1} (0, 1, 10, 25, 50, 75 and 100 µg mL^{-1})

Concentration (µg mL^{-1})	Volume of Saltwater (µL)	Volume of $K_2Cr_2O_7$ Stock Solution (µL)	Total Volume (µL)
0 (Control)	5000	0	5000
1	4960	40	5000
10	4600	400	5000
25	4000	1000	5000
50	3000	2000	5000
75	2000	3000	5000
100	1000	4000	5000

5. Representative image of *Artemia salina* in toxicity test

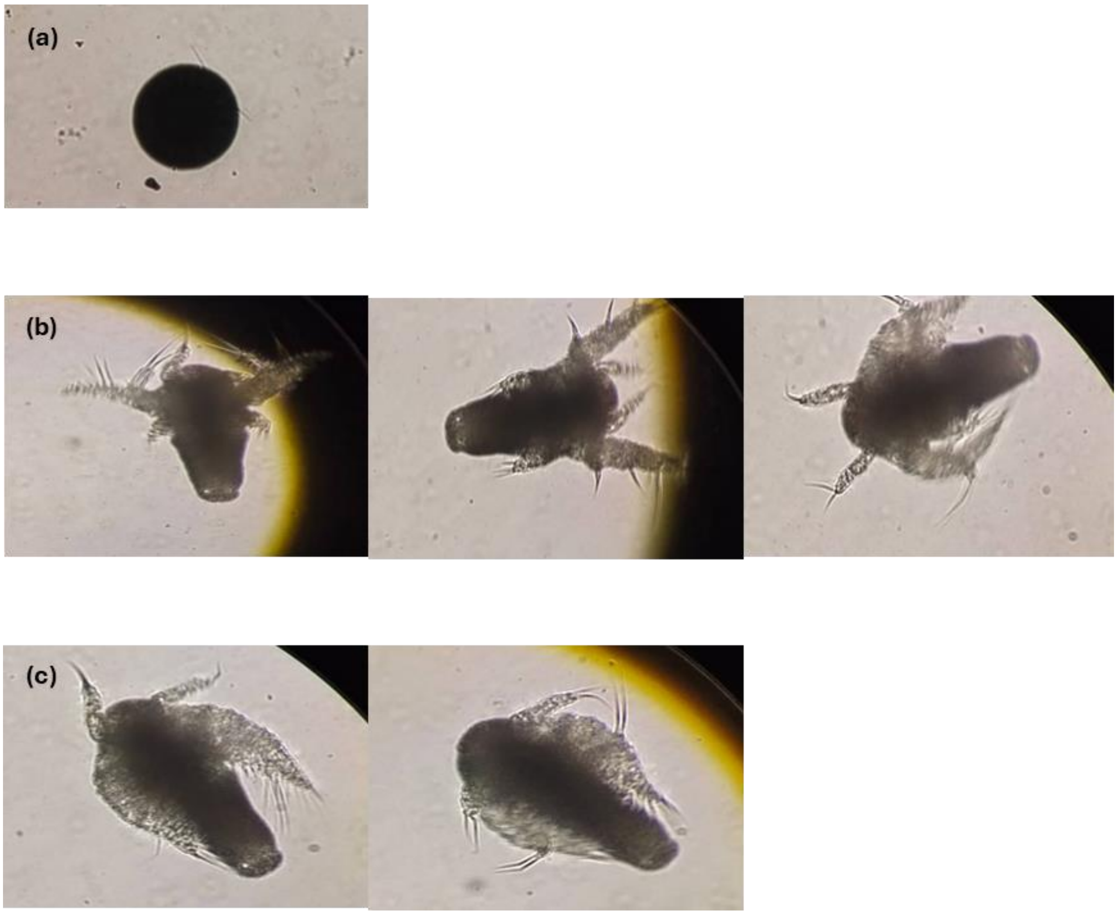

Supplementary Figure S6. Selected icons of (a) hatching egg, (b) alive and (c) dead *Artemia salina* nauplii which were treated with the sample, SnO_2 NPs and standard, $K_2Cr_2O_7$.

REFERENCES

1. Vidya, C., Manjunatha, C., Chandraprabha, M. N., Rajshekar, M., & MAL, A. R. (2017). Hazard free green synthesis of ZnO nano-photo-catalyst using Artocarpus Heterophyllus leaf extract for the degradation of Congo red dye in water treatment applications. *Journal of Environmental Chemical Engineering*, *5*(4), 3172-3180.
